# Porcine transient receptor potential channel 1 promotes adipogenesis and lipid deposition

**DOI:** 10.1016/j.jlr.2024.100718

**Published:** 2024-12-03

**Authors:** Yu Fu, Xin Hao, Jingru Nie, Peng Shang, Xinxing Dong, Bo Zhang, Dawei Yan, Hao Zhang

**Affiliations:** 1 Frontiers Science Center for Molecular Design Breeding (MOE), China Agricultural University, Beijing, China; 2 State Key Laboratory of Animal Biotech Breeding, China Agricultural University, Beijing, China; 3 College of Animal Science, Xizang Agricultural and Animal Husbandry College, Linzhi, China; 4 College of Animal Science and Technology, Yunnan Agricultural University, Kunming, China

**Keywords:** porcine, TRPC1, adipogenesis, lipid deposition, PI3K/AKT, β-catenin

## Abstract

Adipose tissue, an important organ involved in energy metabolism and endocrine, is closely related to animal meat quality and human health. Transient receptor potential channel 1 (TRPC1), an ion transporter, is adipocytes’ major Ca^2+^ entry channel. However, its function in fat deposition is poorly understood, particularly in pigs, which are both an ideal model for human obesity research and a primary meat source for human diets. In the present investigation, our findings demonstrate a prominent expression of TRPC1 within the adipose tissue of pigs with a strong fat deposition ability. Functional analysis showed that *TRPC1* promotes primary preadipocyte proliferation and adipogenic differentiation. In vivo, transgenic mice expressing porcine *TRPC1* exhibited aggravated high-fat diet–induced obesity, hepatic steatosis, and insulin resistance. Moreover, TRPC1 may facilitate adipogenesis via activating phosphatidylinositol 3 kinase/AKT and β-catenin signaling pathways. Our research underscores the pivotal role of porcine *TRPC1* as a positive regulator in adipogenesis and lipid accumulation processes, providing a potential target for improving animal meat quality and treating obesity-related diseases in humans.

## Graphical abstract

**Figure:**
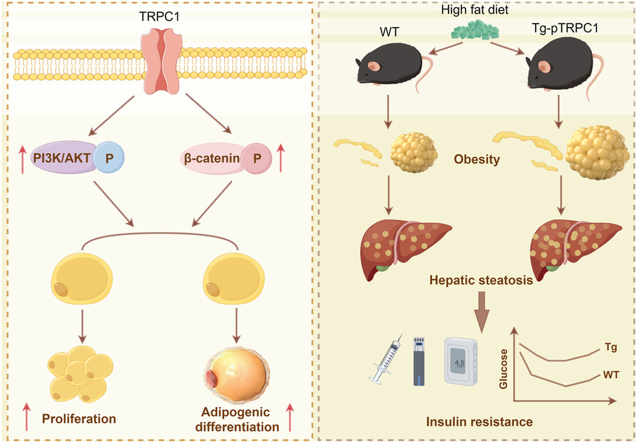

Fat is a multifunctional tissue that is significant in balancing energy equilibrium within the body and insulin sensitivity; however, its excessive accumulation can lead to obesity, inflammation, and other metabolic diseases (1). White adipose tissue (WAT), the largest energy storage depot in adults, primarily converts excess energy into triglycerides for storage and provides energy to other tissues when required (2). Moderate amounts of fat contribute to organ protection and animal meat’s quality and nutritional value. However, excessive accumulation of WAT can lead to obesity, which not only has a negative impact on important economic traits, such as growth and reproductive performances and meat quality in livestock and poultry (3), but also seriously impairs overall metabolic health and elevates the possibility of developing cardiovascular disease, diabetes, and certain cancers in humans (4). Therefore, understanding the processes and regulatory pathways of adipogenesis can help formulate reasonable strategies to control animal fat deposition and treat diseases related to human fat metabolism. The augmentation of both the size and quantity of adipocytes causes fat deposition. Adipogenesis refers to the process in which preadipocyte differentiation into mature adipocytes and includes cell proliferation and differentiation (5). This intricate biological process is regulated by multiple factors, making it important to explore these genes and their corresponding regulatory networks.

Transient receptor potential channel 1 (*TRPC1*) encodes a nonselective cation channel pivotal in regulating intracellular calcium homeostasis and cell proliferation, apoptosis, migration, and differentiation (6, 7, 8). *TRPC1* is highly expressed in differentiated myocytes (9) and main metabolic tissues, including adipose tissue (10), brain (11), and skeletal muscle (12), suggesting a potential role in regulating energy metabolism and adiposity. TRPC1 is the major Ca^2+^ entry channel in adipocytes, and its absence destroys Ca^2+^ and triacylglycerol homeostasis (10, 13). *TRPC1* knockdown or knockout reduces adipocyte differentiation and the expression level of metabolic genes (9, 14). Sukumar *et al.* reported that *TRPC1* is upregulated during WAT maturation and negatively regulates adiponectin, which confers insulin sensitization effects (15). In vivo *TRPC1* deficiency results in reduced placental, fetal, and adult weights (16, 17). Moreover, *TRPC1*^−/−^ mice were fed a high-fat diet (HFD). They exercised and reduced their risk of developing diet-induced type II diabetes and obesity, evidenced by decreased fat mass, insulin resistance (IR), and adipocyte numbers compared to those in control mice (10). Taken together, the phenotypes described above are observed based on the loss-of-function of channels in *TRPC1*^−/−^ mice, indicating an important function of *TRPC1* in modulating fat deposition. However, the underlying intricate mechanism remains unclear. Overexpression of *TRPC1* may further explore its biological function and regulatory pathways through forward genetics. *Sus scrofa* is an important agricultural animal highly similar to humans in its physiology, anatomy, pathology, and genetic regulation of fat deposition (18, 19, 20). Exploring the previously unreported function of porcine *TRPC1* in fat deposition may contribute to improvements in agriculture and the treatment of fat-related diseases.

Here, we analyzed the expression level of *TRPC1* in the fat tissues of pigs with different fat deposition abilities. Primary preadipocytes were used in vitro to explore the function of porcine *TRPC1* in cell proliferation and lipogenic differentiation. In vivo, transgenic miceexpressing porcine *TRPC1 (Tg-pTRPC1)* on a HFD were used as obesity models to explore the potential effect of *TRPC1* on fat deposition, hepatic steatosis, glucose metabolism, and insulin sensitivity. Moreover, we identified potential targets of fat deposition mediated by TRPC1. Our research reveals that TRPC1 is critical for adipogenesis and can improve animal meat quality and the treatment of human obesity.

## Materials and methods

### Experimental animals

Two native Chinese pig breeds, Tibetan pigs and Wujin pigs, and a lean-type pig breed, Yorkshire pigs (YY), were reared on standard diets and water at the Xizang Agriculture and Animal Husbandry University Farm, Linzhi, Xizang. Back fat tissues were collected from embryos and 6-month-old pigs. Embryonic samples were obtained from the embryos of two pregnant sows 60 days postinsemination. Animal feeding and testing were conducted according to the National Research Council Guide for the Care and Use of Laboratory Animals, with the experimental protocols approved by the Institutional Animal Care and Use Committee at China Agricultural University (permit number: AW80203202-1-1).

### Primary preadipocytes isolation

Primary preadipocytes were aseptically isolated from the subcutaneous tissue of 6-week-old mice and 7-day-old YY pigs. Subcutaneous adipose tissues were minced and dissociated with 2 mg/ml collagenase type I (Sigma-Aldrich) at 37°C for 40 min in a reciprocating shaker bath, followed by filtration through a 70 μm filter membrane (Biosharp, Guangzhou, China). The preadipocytes were centrifuged and suspended in DMEM (Gibco, Grand Island, NY) supplemented with 10% FBS (Gibco).

### Cell culture and preadipocyte differentiation

Primary preadipocytes were cultured in a complete medium (DMEM with 10% FBS and 1% penicillin-streptomycin) at 37°C in a 5% CO_2_ incubator. Fully confluent preadipocytes were differentiated in DMEM supplemented with 1 μM dexamethasone, 0.5 mM 1-methyl-3-isobutylxanthine, and 5 μg/ml insulin for 2 days (all from Sigma-Aldrich, St. Louis, MO). The medium was maintained with 5 μg/ml insulin and replaced every 2 days.

### RNA extraction and quantitative real-time PCR

RNA extraction was performed with the TRIzol reagent (Invitrogen, Carlsbad, CA). Total RNA (2 μg) was reversed-transcribed into cDNA using reverse transcriptase (TIANGEN, Beijing, China), and the SYBR Mix (TIANGEN) was used for quantitative real-time PCR. The mRNA levels of the target gene were standardized relative to *β-actin* by referring to the 2^-ΔΔCt^ method (21). Primer sequences used for quantitative real-time PCR are provided in the supplemental information (supplemental Table S1).

### Interference fragment synthesis, plasmid construction, and transfection

siRNAs targeting porcine *TRPC1* were synthesized by Genepharma (Genepharma, Shanghai, China); all siRNA sequences are presented as described in supplemental Table S2. To overexpress the *TRPC1*, the full-length sequence of porcine-*TRPC1* was cloned into the pcDNA3.1 plasmid (Addgene) using specific F/R primers (F: 5′-ATGATGGCGGCCCTGTACCC-3’; R: 5′-TTAATTTCTTGGATAAAACA-3′). The primary preadipocytes were transfected using Lipofectamine 2000 (Invitrogen).

### Cell proliferation assays

Cell proliferation was analyzed using 5-ethynyl-2′-deoxyuridine (EdU) staining (Ribobio, Guangzhou, China) and Cell Counting Kit-8 (CCK-8, Beyotime Biotechnology, Shanghai, China) assays. After mixing and counting, approximately 1∗10^4^ cells per well were seeded into 96-well plates. Transfection was performed when the cell confluence reached 40%–60%, and cell proliferation was assessed 24 h after transfection. The transfected cells were cultured in DMEM supplemented with 50 mM EdU at 37°C for 2 h. Subsequently, the fixed cells were permeabilized with 0.5% Triton X-100 to allow the EdU detection reagent to enter the cells. The cells were incubated in the Apollo reaction solution for 1 h and 4',6-diamidino-2-phenylindole for 30 min. Images of EdU-labeled cells were captured using a fluorescence microscope (Q500 MC, Leica, Wetzlar, Germany) to evaluate cell viability. For the CCK-8 assay, cells were incubated in a DMEM with 10% CCK-8 at 37°C for 1 h in the dark, followed by measurement of the absorbance at 450 nm using a microplate reader (Biotek, Winooski, VT).

### Oil Red O and BODIPY staining

The accumulation of lipid droplets was used to denote adipogenic differentiation. For cell differentiation experiments, approximately 3∗10^5^ cells per well were evenly seeded into 24-well plates through cell counting, and transfection was performed once the cells reached 90% confluence. Six hours posttransfection, the cells reached near full confluence; at this point, we replaced the growth medium with a differentiation medium to induce differentiation. After fixation in 4% paraformaldehyde, differentiated adipocytes or tissues were washed with 60% isopropanol for 10 s, stained with Oil Red O (Sigma-Aldrich) for 30 min, and photographed. Stained Oil Red O was extracted in 100% isopropanol, and absorbance was measured at 510 nm using a spectrophotometer (Biotek, Winooski, VT). BODIPY stock solution (Sigma-Aldrich) was diluted to a working concentration of 1:1000 in PBS (Gibco). The fixed cells were incubated with BODIPY staining solution for 30 min. PBS was used to wash and remove any unbound BODIPY dye and reduce background staining. 4',6-diamidino-2-phenylindole was used for nuclear counterstaining. Visualization and capture of BODIPY fluorescent signals were performed using a fluorescence microscope (Leica).

### Generation of *Tg-pTRPC1* and phenotype measurements

The Rosa26 site positioned on chromosome 6 is a highly favored location among those utilized to integrate foreign DNA (22). Based on the CRISPR/Cas9 technology, we generated Tg-p*TRPC1* in intron1 of the Rosa26 locus within the C57BL/6J genetic background. Two guide RNAs (gRNA1: 5′-GGCAGGCTTAAAGGCTAACC-TGG-3’; gRNA2: 5′-CTCCAGTCTTTCTAGAAGAT-GGG-3′) were designed using the online website (http://tools.genome-engineering.org). WT and Tg mice were validated by PCR with specific primers (WT-F: 5′-CACTTGCTCTCCCAAAGTCGCTC-3’; WT-R: 5′-ATACTCCGAGGCGGATCACAA-3’; Tg-F: 5′-GGCAACGTGCTGGTTATTGTG-3’; Tg-R: 5′-CCATATAATAGTCACCCTTGTCG-3′). WT and Tg mice showed amplicon sizes of 453 bp and 244 bp, respectively. WT and Tg offspring mice of both genders were randomly selected, and their body, adipose, and organ weights were measured. The mice were divided into the normal feeding diet and HFD groups. The HFD group was fed a HFD from 2 to 5 months of age.

### Histology staining

Fat and liver samples from HFD-fed mice were fixed, dehydrated, and embedded in paraffin. The paraffin-embedded tissue sections were stained with H&E solution. Finally, the sealed sections were placed under a microscope (ZEISS, Jena, Germany) to observe the tissue morphology and cell characteristics.

### Glucose and insulin tolerance tests

HFD-fed mice aged 20-week-old were used to perform the glucose tolerance test (GTT) and insulin tolerance test (ITT) as previously described (23). Intraperitoneal injections of 20% glucose (2 g/kg body weight) or insulin (0.75 U/kg body weight) were administered after 16 and 4 h of fasting, respectively. Whole blood samples were collected from the tail vein at zero time points for baseline glucose measurements. Whole blood glucose samples were taken for the GTT at 15, 30, 60, 90, and 120 min via tail bleeding. Blood samples were collected for the ITT at 15, 30, 45, 60, 90, and 120 min.

### Western blot

Tissues or cells transfected for 48 h were used for Western blot assays. The samples were lysed and centrifuged, and the supernatant was collected to obtain a protein solution. Proteins were loaded onto 10% SDS-PAGE gels, transferred to membranes, and blocked. The protein bands were visualized using the chemiluminescence kit after incubation with specific primary and secondary antibodies, followed by horseradish peroxidase–conjugated antibodies (UElandy, Suzhou, China). The primary antibodies used were as follows: anti-TRPC1 (ab51255, 1:1000, Abcam, Cambridge), anti-PPARγ (sc7273, 1:200, Santa, NC), anti-CEBPα (D56F10, 1:1000, CST, Danvers, MA), anti-β-actin (4970, 1:1000, CST), anti-AKT (9272, 1:1000, CST), anti-p-AKT-Ser473 (4060, 1:1000, CST), anti-phosphatidylinositol 3 kinase (PI3K) (4249, 1:1000, CST), anti-p-PI3K (13,857, 1:1000, CST), anti-β-catenin (ab16051, 1:1000, Abcam), and anti-p-βcatenin (AP0579, 1:2000. Abclonal).

### Magnetic resonance imaging

MRI was performed at the Institute of Laboratory Animals Science, CAMS & PUMC. Mice fed a HFD were anesthetized with 2% isoflurane, and the whole-body fat was scanned using MRI. The images were analyzed using vnmrj4.0 software.

### RNA-seq and functional annotation

WT and Tg primary adipocytes were subjected to transcriptome sequencing after 4 days of differentiation, with three biological replicates for each group. TRIzol RNA Reagent (Takara, Dalian, China) was used to extract total RNA, as described above. The Illumina Hiseq-Xten platform (Frasergen Information Co., Ltd., Wuhan, China) was utilized to construct a cDNA library and the subsequent execution of high-throughput sequencing. The differentially expressed genes (DEGs) were screened using the criterion |log2 (fold change)| > 2 and P-adjusted ≤ 0.01, and performed function annotation of Gene Ontology and Kyoto Encyclopedia of Genes and Genomes pathways using the DAVID online software (http://david.abcc.ncifcrf.gov/home.jsp).

### Statistical analysis

Data are presented as means ± SD. A one-way ANOVA with Tukey’s posthoc test was employed to analyze the statistical significance of comparisons across multiple groups. In contrast, a Student’s *t* test was utilized for pairwise comparisons between the two groups. *P* < 0.05 was considered to be statistically significant. The data are representative of three or more independent experiments. All analyses were performed using SPSS software 17.0.

## Results

### TRPC1 positively regulates the proliferation and differentiation of porcine preadipocyte

**Fig. 1:**
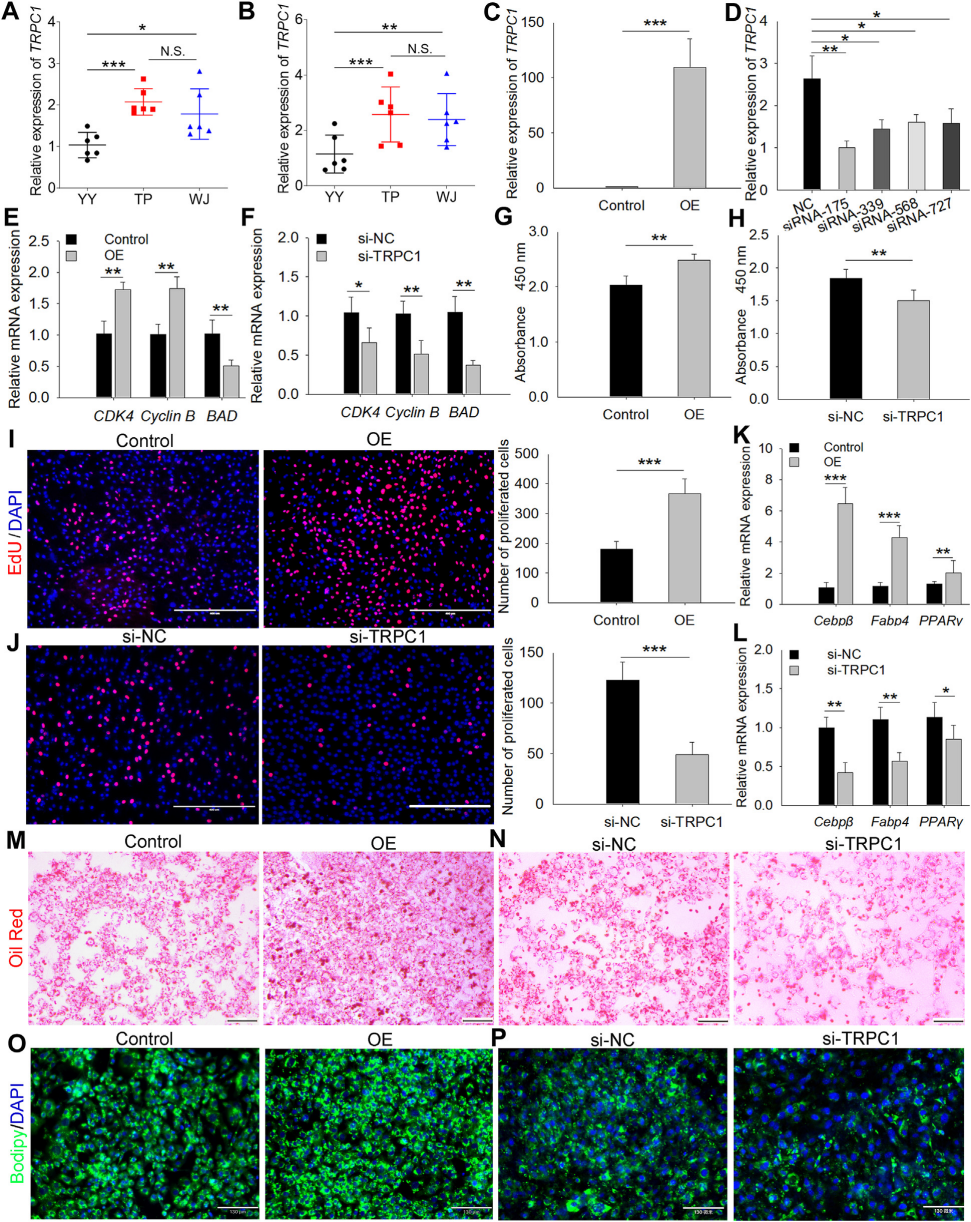
*TRPC1* is involved in fat deposition in pigs. *TRPC1* mRNA expression levels in back fat tissue of 60-day-old embryos (A) and 6-month-old (B) pigs. YY, Yorkshire (n = 6); TP, Tibetan pig (n = 6); and WJ, Wujin pig (n = 6). Efficiency of the overexpression (OE) plasmid (C) and RNA interference fragments (D). NC indicates negative control. mRNA expression levels of proliferation-related genes (*CDK4* and *Cyclin B*) and apoptotic gene (*BAD*) in *TRPC1* overexpression (E) and knockdown (F) cells. Cell Counting Kit-8 (CCK-8) assay of proliferating porcine preadipocytes transfected with *TRPC1* overexpression (G) or interference (H) fragments. 5-ethynyl-2′-deoxyuridine (EdU) staining of proliferating cells after pcDNA3.1-*TRPC1* (I) or si-*TRPC1* (J) transfection. Nuclei were stained with DAPI, n = 3 in each group, scale bar = 400 μm. mRNA expression of adipogenic differentiation marker genes (*CEBPβ*, *Fabp4*, and *PPARγ*) in *TRPC1* overexpression (K) and knockdown (L) cells. Oil Red O staining of pcDNA3.1-*TRPC1* (M) or si-*TRPC1* (N)-treated cells cultured for 4 days in a differentiation medium. Red indicates fat droplets formed by differentiation, scale bar = 200 μm. BODIPY staining of differentiated cells with *TRPC1* overexpression (O) or knockdown (P). Green represents BODIPY-labeled fat droplets, and blue indicates DAPI-labeled nuclei, scale bar = 130 μm. Each bar represents the means ± SD of three independent experiments. ∗: *P* < 0.05, ∗∗: *P* < 0.01, and ∗∗∗: *P* < 0.001. DAPI, 4',6-diamidino-2-phenylindole; CEBP, CCAAT/enhancer-binding protein; Fabp4, fatty acid–binding protein 4; TRPC1, transient receptor potential channel 1.

We measured the expression level of *TRPC1* in the back fat samples of 60-day-old embryos and 6-month-old pigs. We found that Tibetan pigs and Wujin pigs, which have strong fat deposition ability, had significantly higher expression than in YY pigs, which have low-fat deposition ability (Fig. 1A, B). These indicated that the expression level of *TRPC1* may be positively associated with fat deposition in pigs. To investigate the function of *TRPC1*, primary porcine preadipocytes were isolated and transfected with overexpression plasmids (Fig. 1C) and RNA interference fragments (Fig. 1D). We synthesized four interference fragments for porcine *TRPC1* and selected the siRNA-175 with the best interference efficiency for subsequent study (Fig. 1D). The mRNA level of key proproliferation genes (*CDK4* and *Cyclin B*) was evaluated and the apoptosis gene *BAD* was significantly downregulated after overexpression of *TRPC1* (Fig. 1E); *TRPC1* knockdown group showed the opposite expression trend (Fig. 1F). CCK-8 and EdU assays showed that *TRPC1* overexpression increased the absorbance value (Fig. 1G) and number of proliferated cells (Fig. 1I). When *TRPC1* was knocked down, the absorbance (Fig. 1H) and EdU incorporation (Fig. 1J) were decreased. Additionally, the expression of lipogenic differentiation markers (CCAAT/enhancer-binding protein beta, *CEBPβ*; fatty acid–binding protein 4, *Fabp4*; and *PPARγ*) were observably elevated after *TRPC1* overexpression (Fig. 1K), and *TRPC1* knockdown inhibited their expression (Fig. 1L). Meanwhile, Oil Red and BODIPY staining revealed that more fat droplets were differentiated in the *TRPC1* overexpressed cells compared to the control group (Fig. 1M, O), and *TRPC1* interference decreased the formation of fat droplets (Fig. 1N, P). All these observations imply that *TRPC1* plays a positive role in preadipocyte proliferation and differentiation in pigs.

### Tg-pTRPC1 increases HFD-induced obesity

**Fig. 2:**
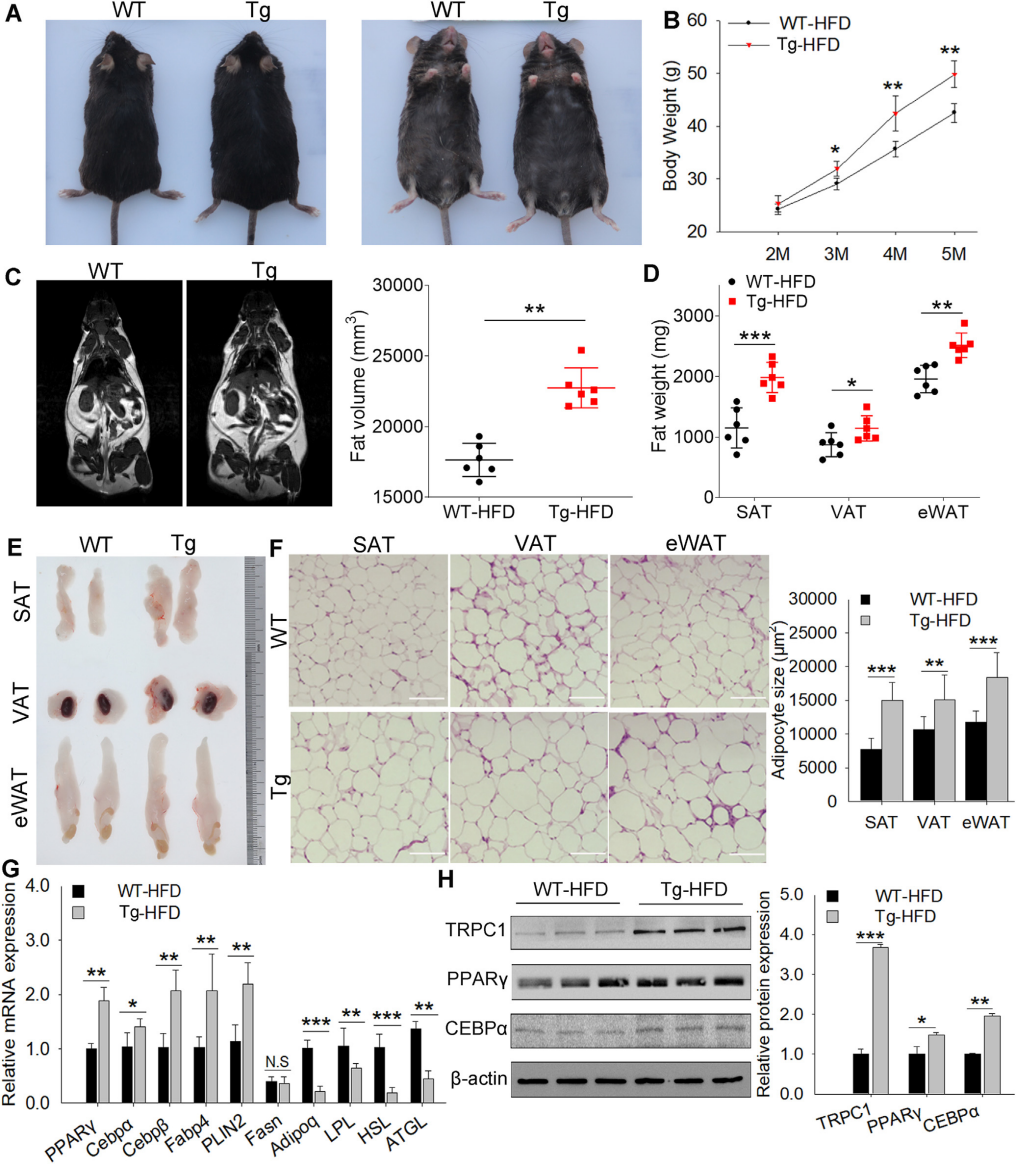
Tg-p*TRPC1* increases high-fat diet (HFD)-induced obesity. Representative gross morphology (A) and body weight curves (B) of whole bodies of WT and transgenic (Tg) mice at 20 weeks of age fed an HFD for 12 weeks; n > 10. C: Representative magnetic resonance images of whole fat of WT and Tg mice. Fat volumes are presented as mm^3^ (n = 6). D: Quantification of fat weight in HFD-fed mice (n = 6). E: Representative images of fat pads of HFD-fed mice. F: Representative images of H&E-stained SAT, VAT, and eWATsections from HFD-fed mice; scale bar = 210 mm. G: mRNA expression level of lipogenic-related genes (*PPARγ*, *CEBPα, CEBPβ*, *FABP4, PLIN2*, and *Fasn*) and lipolysis-related genes (*Adipoq, LPL, HSL, and ATGL*) in fat tissues of HFD fed mice, n = 6. H: Western blot analysis of PPARγ and CEBPα levels in fat tissues of HFD-fed mice, n = 6. The data represent the mean ± SD. ∗: *P* < 0.05, ∗∗: *P* < 0.01, and ∗∗∗: *P* < 0.001. Adipoq, adiponectin; ATGL, adipose triglyceride lipase; CEBP, CCAAT/enhancer-binding protein; eWAT, epididymal white adipose tissue; Fabp4, fatty acid–binding protein 4; Fasn, fatty acid synthase; HSL, hormone-sensitive lipase; LPL, lipoprotein lipase; N.S., not significant; PLIN2, perilipin 2; SAT, subcutaneous adipose tissue; TRPC1, transient receptor potential channel 1; VAT, visceral adipose tissue.

We generated Tg-p*TRPC1* to explore the in vivo function of porcine TRPC1 in fat deposition. As shown in supplemental Fig. S1A, the expression level of porcine *TRPC1* increased in Tg mice, whereas Tg and WT mice showed similar expression levels of mouse *TRPC1*. Under normal feeding diet conditions, the weights of subcutaneous, visceral, and epididymal white adipose tissues (subcutaneous white adipose tissues, visceral white adipose tissues, and epididymal white adipose tissues) were higher in Tg mice aged 2–4 months of age than in WT mice, even though both groups were healthy and born at the expected Mendelian ratio (supplemental Fig. S1B–D). However, the weight of the interscapular brown adipose tissue did not differ significantly between WT and Tg mice (supplemental Fig. S1B–D). Therefore, we studied the function of *TRPC1* in the development of white fat. To better understand *TRPC1* function in vivo, we subjected the mice to a HFD to induce obesity. Compared to the WT mice, Tg mice displayed increased body weight (Fig. 2A, B) but no significant difference in food intake (supplemental Fig. S1E). Analysis of fat mass by MRI and quantification of fat weight revealed increased body adiposity in Tg mice compared to WT mice (Fig. 2C–E). Histological analysis revealed that the adipocyte area in the adipose tissue of Tg mice was larger than that in the adipose tissue of WT mice (Fig. 2F). Additionally, the mRNA expression level of the adipocyte differentiation- and lipogenic-related genes PPARγ, *CEBPα, CEBPβ*, *FABP4*, and *perilipin 2* (*PLIN2*) were markedly upregulated in the adipose tissue of Tg mice, whereas the expression of fatty acid synthase was not significantly different between groups (Fig. 2G). In contrast, the mRNA expression level of the lipolysis-related genes adiponectin (*Adipoq*), lipolysis (lipoprotein lipase), hormone-sensitive lipase, and adipose triglyceride lipase were significantly downregulated (Fig. 2G). The PPARγ and CEBPα protein levels were consistent and corresponded to the mRNA levels (Fig. 2H). These results indicated that *TRPC1* positively regulates fat deposition in mice, which is conserved in pigs.

### Tg-pTRPC1 displayed aggravated hepatic steatosis and IR

**Fig. 3:**
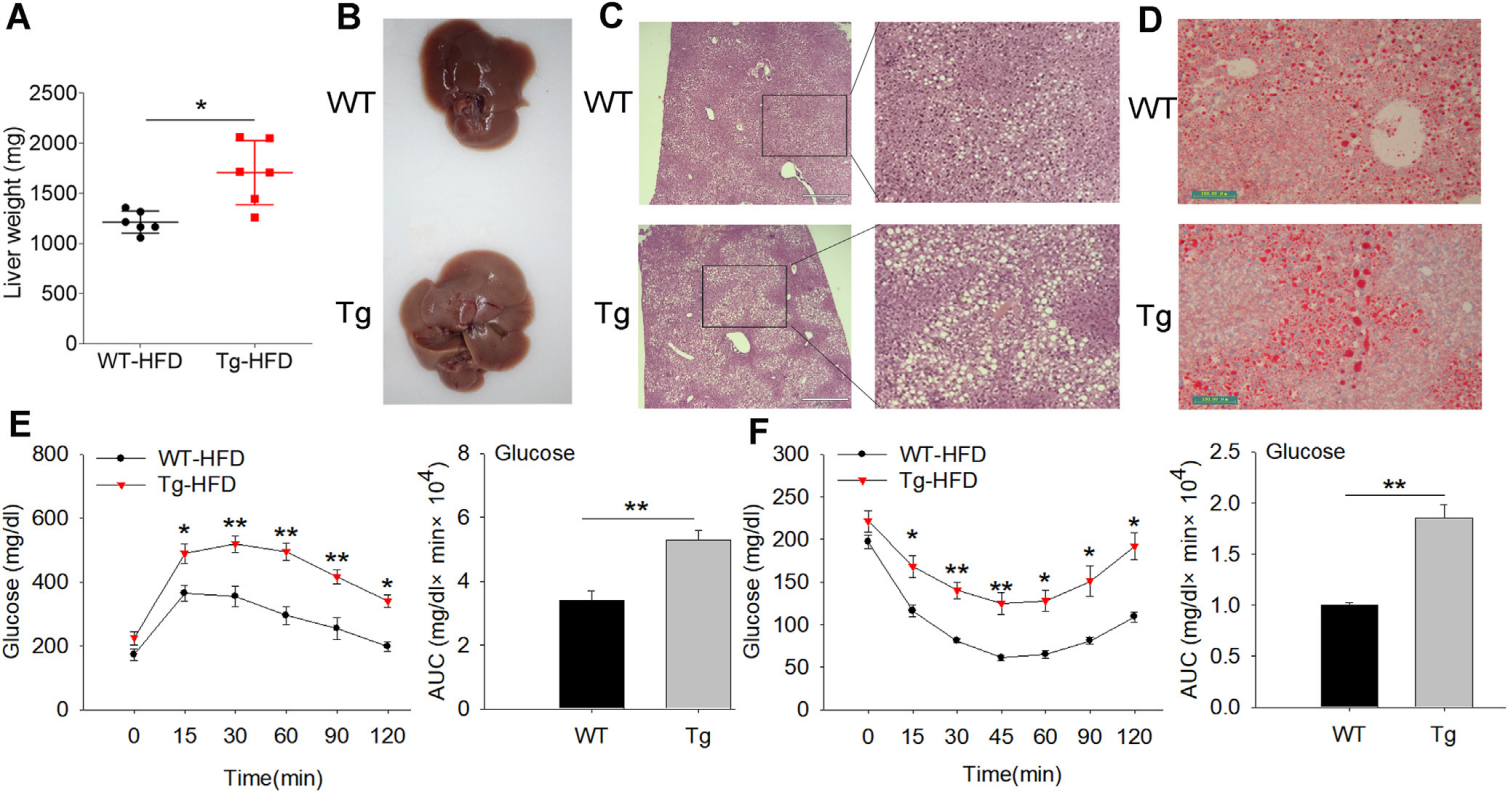
Tg-p*TRPC1* aggravates HFD-induced hepatic steatosis and IR. A: Liver weight of HFD-fed WT and Tg mice at 20 weeks of age fed an HFD for 12 weeks (n = 6). Representative images (B) and sections of the liver stained with H&E (C) and Oil Red O (D). H & E, scale bar = 210 μm; Oil Red O, scale bar = 100 μm. Glucose tolerance (E) and insulin tolerance (F) tests in HFD-fed mice (n > 4). Bar charts show the area under the curve (AUC) for glucose. The data represent the mean ± SD. ∗: *P* < 0.05 and ∗∗: *P* < 0.01. HFD, high-fat diet; IR, insulin resistance; Tg-pTRPC1, transgenic mice expressing porcine TRPC1; TRPC1, transient receptor potential channel 1.

The liver is well known as the center of fat metabolism and transportation, capable of synthesizing and storing lipids to supply the whole body’s needs. Interestingly, we found that the livers of Tg mice were heavier, larger, and slightly whiter than those of WT mice (Fig. 3A, B). Further histological analysis revealed that hepatocyte lipid droplets were higher in Tg mice than in WT mice (Fig. 3C, D), suggesting hepatic fat deposition. Obesity is often associated with reduced glucose metabolism and insulin sensitivity, which increase the risk of diabetes. We performed GTT and ITT by measuring blood glucose levels. The GTT results showed that the glucose levels in both groups rapidly increased. Then, with a gradual decrease after glucose injection, the glucose concentration and area under the curve in the Tg group were markedly higher than those in the WT group, indicating that glucose intolerance existed in Tg mice (Fig. 3E). The ITT showed reduced insulin sensitivity in Tg mice, as demonstrated by higher glucose levels and area under the curve after insulin injection than in WT mice (Fig. 3F). Blood biochemical results showed that compared with WT, the levels of blood glucose, insulin, lipid profile, leptin, TNF-α, IL-6, and resistin were significantly elevated in Tg mice (Table 1). These results revealed that Tg-p*TRPC1* showed aggravated heterotopic lipid deposition and IR during HFD-induced obesity.

**Table 1:** Serum metabolite and adipokine levels in HFD-fed mice

Parameters	WT	Tg	*P* Value
Glucose metabolism			
Glucose (mmol/l)	4.86 ± 0.61	6.82 ± 1.70	0.03^a^
Insulin (uIU/ml)	11.53 ± 1.34	14.60 ± 4.18	0.01^a^
Lipid profile			
Total cholesterol (mmol/l)	1.14 ± 0.14	1.51 ± 0.15	0.002^b^
Triglyceride (mmol/l)	0.19 ± 0.02	0.29 ± 0.07	0.04^a^
Nonesterified fatty acid (mmol/l)	0.36 ± 0.03	0.39 ± 0.07	0.46
High-density lipoprotein (mmol/l)	0.72 ± 0.09	0.87 ± 0.09	0.003^b^
Low-density lipoprotein (mmol/l)	0.13 ± 0.03	0.28 ± 0.06	0.01^a^
Adipokine levels			
Leptin (ng/ml)	3.81 ± 0.30	4.44 ± 0.93	0.03^a^
TNF-α (pg/ml)	53.11 ± 12.90	71.87 ± 6.45	0.04^a^
Interleukin 6 (pg/ml)	100.19 ± 26.06	132.35 ± 6.69	0.03^a^
Resistin (ng/ml)	17.99 ± 5.75	26.15 ± 2.45	0.04^a^
Adiponectin (mg/ml)	14.17 ± 0.45	13.27 ± 1.40	0.53

HFD, high-fat diet.

a *P* < 0.05.

b *P* < 0.01. Data are represented as mean ± SD. n = 8 animals per group.

### TRPC1 promotes the proliferation and differentiation of mouse primary preadipocytes

**Fig. 4:**
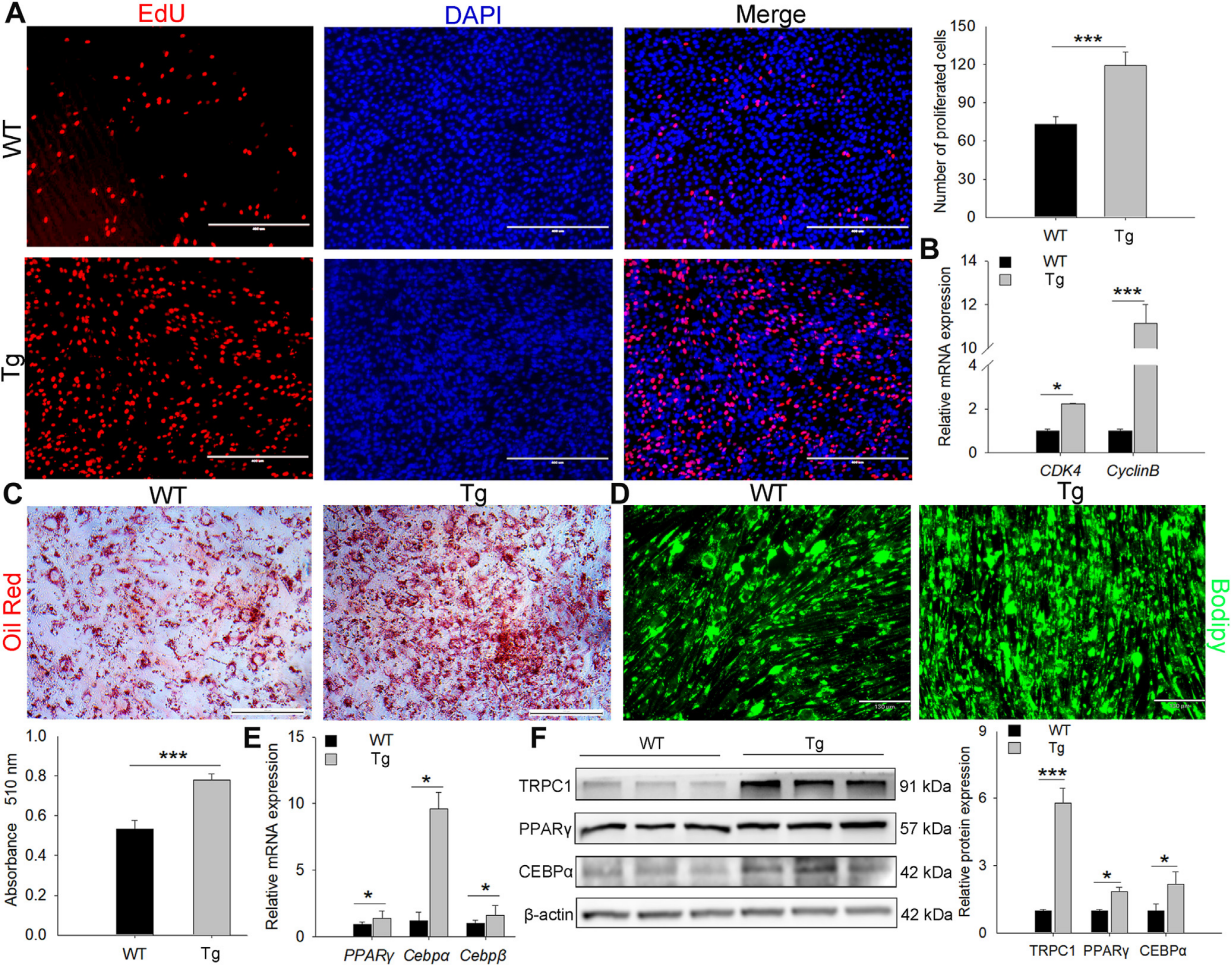
*TRPC1* facilitates the proliferation and differentiation of mouse primary preadipocytes. A: Cell proliferation was assessed by EdU staining. DAPI-stained nuclei are shown (scale bar = 400 μm). B: mRNA levels of *CDK4* and *Cyclin B* (proliferation markers) were quantified by qRT-PCR. C: Representative images of Oil Red O staining in control and Tg cells allowed to differentiate for 4 days. Oil Red O–stained fat droplets are shown in red. Oil Red O–labeled fat droplets were eluted with isopropyl alcohol, and absorbance was measured at 510 nm (lower bar chart). D: BODIPY staining of differentiated WT and Tg adipocytes. Green represents the BODIPY-labeled fat droplets. mRNA (E) and protein (F) expression levels of adipogenic differentiation marker genes in mouse primary preadipocytes. Data represent mean ± SD, n = 3. ∗: *P* < 0.05 and ∗∗∗: *P* < 0.001. DAPI, 4',6-diamidino-2-phenylindole; EdU, 5-ethynyl-2′-deoxyuridine; qRT-PCR, quantitative real-time PCR; TRPC1, transient receptor potential channel 1.

The primary mouse preadipocytes were isolated to investigate the biological functions of *TRPC1*. Compared to the WT, preadipocytes from the Tg group showed increased proliferation and expression of proproliferation marker genes, suggesting that TRPC1 promotes preadipocyte proliferation (Fig. 4A, B). Oil Red and BODIPY staining revealed more fat droplets in the Tg cells than in the WT cells (Fig. 4C, D). Tg-p*TRPC1* also enhanced lipogenic differentiation markers’ mRNA and protein levels (Fig. 4E, F). These findings on *TRPC1* promoting the proliferation and differentiation of mouse preadipocytes are consistent with previous results in pig cells, revealing that the biological function of *TRPC1* in adipocytes may be conserved in pigs and mice.

### TRPC1 facilitates adipogenesis via phosphorylation of the PI3K/AKT and β-catenin

**Fig. 5:**
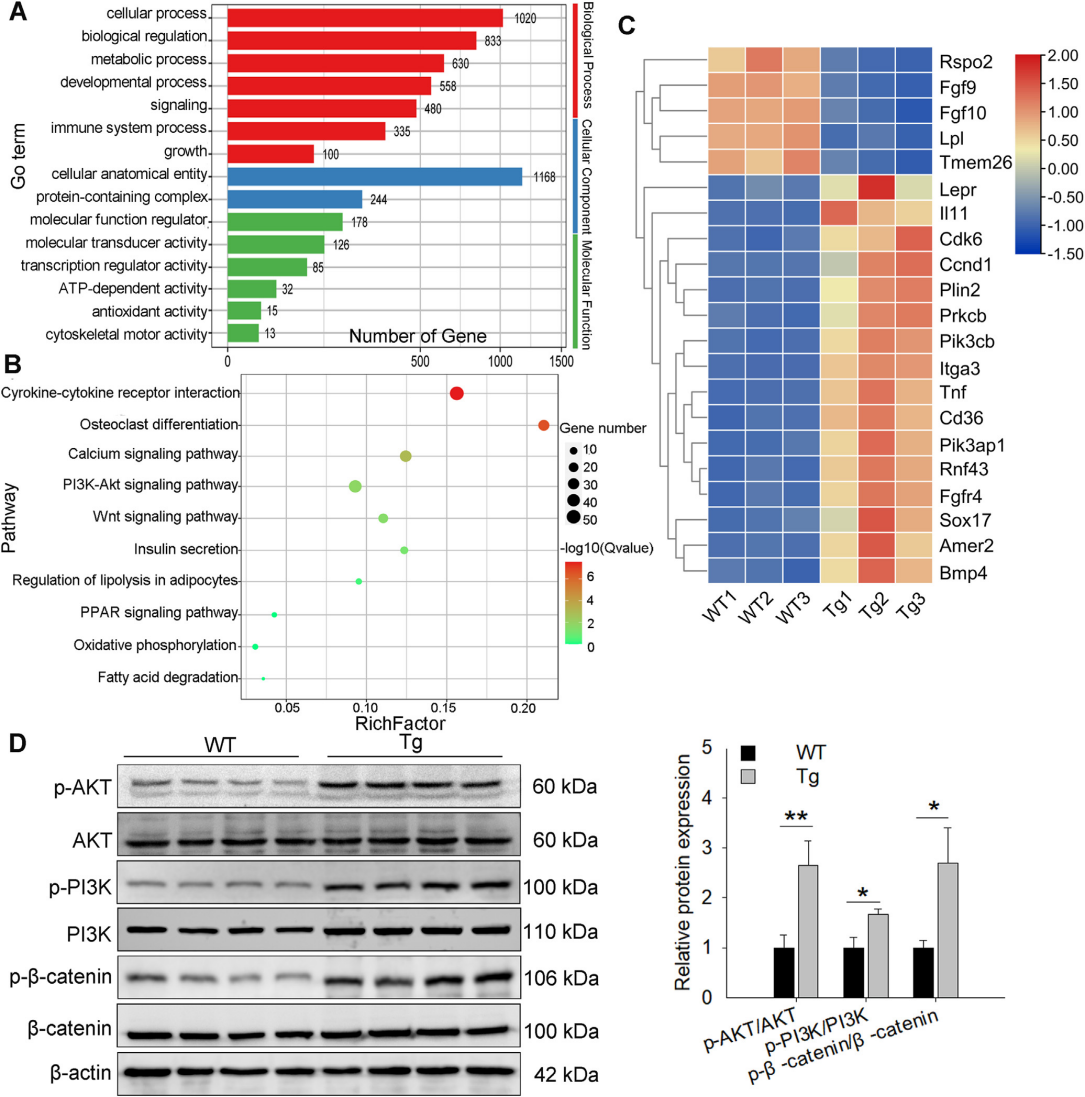
*TRPC1* promotes lipogenesis via the phosphorylation of PI3K/AKT and β-catenin. A: Significantly enriched Gene Ontology terms of differentially expressed genes (DEGs) between WT and Tg mouse primary preadipocytes that were allowed to differentiate for 4 days. Red clusters represent biological processes, blue clusters represent cellular components, and green clusters represent molecular functions of Gene Ontology terms. B: Kyoto Encyclopedia of Genes and Genomes (KEGG)=−enriched scatter plot of DEGs between WT and Tg cells. The rich factor is the ratio of the DEG numbers annotated to the total number of genes annotated in this pathway term. The smaller the *P* value, the higher the significance. C: Heat map analysis of DEGs. D: Western blot analysis of total and phosphorylated PI3K/AKT and β-catenin levels, n = 4. The data represent the mean ± SD. ∗: *P* < 0.05 and ∗∗: *P* < 0.01. PI3K, phosphatidylinositol 3 kinase; TRPC1, transient receptor potential channel 1.

To further explore the pathway of *TRPC1* regulating adipogenesis, RNA sequencing was performed on WT and Tg adipocytes after adipogenic differentiation for 4 days. A total of 1362 DEGs were screened; 876 genes were upregulated, and 486 genes were downregulated in Tg cells (supplemental Fig. S2). The enriched Gene Ontology terms were mainly related to metabolic processes, developmental processes, growth, transcriptional regulator activity, and ATP-dependent activity (Fig. 5A). The representative Kyoto Encyclopedia of Genes and Genomes pathways mainly contained calcium, PI3K/protein kinase B (AKT), Wnt, insulin secretion, lipolysis regulation, PPAR, and oxidative phosphorylation signaling pathways (Fig. 5B). Heat map analysis of DEGs showed that the mRNA expression levels of *Fgf9/10* and lipoprotein lipase were lower in Tg cells than those in WT cells (Fig. 5C). However, some genes were also upregulated in Tg cells, including proliferation (*Cyclin D1, Ccnd1*), lipogenic differentiation (*PLIN2*), PI3K/AKT (*Pik3cb* and *Pik3ap1*), inflammation (tumor necrosis factor, *Tnf*)-related genes, and Amer2 (Fig. 5C). Given the pivotal roles of the PI3K-AKT and Wnt pathways in regulating cellular proliferation and differentiation, we investigated the activation of these pathways in WT and Tg adipocytes and measured the phosphorylation of key proteins. Western blot analysis revealed that *TRPC1* transgenic adipocytes significantly increased the protein expression levels of phosphorylated AKT, PI3K, and β-catenin proteins (p-AKT, p-PI3K, and p-β-catenin), while the expression of total proteins seemed to have no change (Fig. 5D). These findings imply that the promoted lipogenesis phenotype mediated by TRPC1 may stem from its regulation of the PI3K/AKT and Wnt/β-catenin signaling pathways.

## Discussion

Fat, an essential body component, provides energy and maintains metabolic balance, that is, closely related to human health and animal meat quality. Thus, the research on fat deposition processes holds significant importance for both human medicine and agricultural economics. TRPC1, a calcium channel protein, is pivotal in diverse development processes. However, its role in fat deposition is poorly understood, particularly in such economically important agricultural animals as pigs. Here, we observed that *TRPC1* was highly expressed in porcine adipose with a strong adipose deposition capacity, indicating a potential regulatory role for *TRPC1* in lipogenesis.

The proliferation of preadipocytes is an important process in adipocyte maturation. *TRPC1* regulates calcium influx, and *TRPC1* deletion disrupts Ca^2+^ homeostasis, which may lead to mitochondria-mediated apoptosis in adipocytes (10). Porcine adipocytes are an excellent research alternative to human adipocytes and can be used as a model for studying adipocyte proliferation, differentiation, and metabolism (24). We found that *TRPC1* promoted porcine myocyte proliferation and increased the mRNA expression of proproliferation markers. Consistently, *TRPC1* knockdown reduced EdU-positive cells and the mRNA expression levels of proliferation-related genes in mouse myocytes, indicating that the positive regulatory function of *TRPC1* in adipocyte proliferation is conserved in pigs and mice.

Another important feature of mature fat cells is the ability to accumulate lipids. In obesity, fat cells in adipose tissue differentiate and accumulate large fat droplets (25). Dysregulation of adipocyte differentiation gives rise to lipid accumulation and disrupts energy balance and metabolic regulation. Anne Schaar *et al.* found that the expression level of *TRPC1* was upregulated during adipogenic differentiation, and *TRPC1*^−/−^ adipocytes diminished differentiation, as evidenced by reduced lipid accumulation and adipogenic protein expression (9). Similarly, Oil Red O and BODIPY staining showed that *TRPC1* enhanced the aggregation of differentiated lipid droplets during porcine lipogenesis. In the adipogenesis process, some transcription factors play a crucial role, among which PPARγ and CEBPs are the major transcription factors expressed in the early stage of adipogenesis (26, 27, 28, 29, 30). During this process, activated CEBPβ and CEBPδ upregulated the expression of *CEBPα* and *PPARγ*, thereby affecting the expression of many adipocyte marker genes, such as *FABP4*, and promoting the differentiation of preadipocytes into adipocytes (31, 32, 33, 34). Our study found that *TRPC1* knockdown downregulates the mRNA expression levels of *CEBPα*, *FABP4*, and *PPARγ*, further confirming that *TRPC1* potentially promotes the differentiation of preadipocytes into mature adipocytes.

WAT is adults’ main fat type and primarily stores excess energy as lipids, whereas brown adipose tissue primarily dissipates energy as heat (2). We established the Tg-p*TRPC1* model to investigate the relationship between *TRPC1* and obesity. Tg-p*TRPC1* increased WAT weight but had no obvious effect on brown adipose tissue, revealing that *TRPC1* mainly regulates WAT deposition. Tg-p*TRPC1* also increased HFD-induced body weight and fat mass, and there was no significant difference in food intake, revealing that the increase in fat mass in Tg mice is not due to higher energy intake. However, at 13–15 months, *TRPC1*^−/−^ mice displayed increased adipose tissue accumulation compared to WT mice (9); this suggests that *TRPC1* has a complex regulatory mechanism during fat deposition and that this regulation may change with age. Obesity is primarily caused by abnormal lipogenesis and lipolysis. It has been reported that *PLIN2* is accountable for lipid accumulation, and *PLIN2*^−/−^ mice decreased the risk of diet-induced obesity (35, 36). In the current study, the fat tissues of Tg-p*TRPC1* mice showed upregulated expression of lipogenesis markers but downregulated expression levels of lipolysis-related genes. The increase in adipose accumulation could be due to enhanced adipose differentiation and lipogenesis, as well as dysfunctional lipolysis, which, in time, results in hypertrophic expansion in Tg mice. We revealed the role of the *TRPC1* gene in adipogenesis and lipid deposition through tissue expression analysis, cell-level validation, and Tg mouse models. However, the in vivo environment is very complex, and Tg mice with systemic expression of porcine *TRPC1* may affect many cells other than adipocytes, leading to this model’s limitations. In the future, adipose tissue-specific knockout mice can be used to further explore the function of *TRPC1* in adipose development.

When excessive energy is consumed, mature adipocytes in WAT accumulate excessive lipid droplets in the cytoplasm, resulting in adipocyte dysfunction and the metabolic complications of obesity (37). Excess fat accumulates in ectopic tissues such as the liver, the main organ involved in metabolism. It may ultimately lead to metabolic diseases such as nonalcoholic fatty liver disease (38). The Tg mice exhibited increased liver weight and fat deposition capabilities; however, further studies are required to confirm whether they can be diagnosed with nonalcoholic fatty liver disease. These findings may provide new insights and valuable references for studying the pathogenesis and treatment of obesity-induced fatty liver disease and other related conditions.

Moreover, a small adipocyte size enhances insulin sensitivity in animal models (39, 40) and obese and lean humans (41). Our results showed that Tg mice had a larger adipocyte size and a higher lipid profile than WT mice and had reduced glucose tolerance and insulin sensitivity; this is similar to previous findings that *TRPC1*^*−/−*^ mice exhibited exercise-induced suppression of adiposity and diminished IR (10, 15). These phenotypes of aggravated hepatic steatosis and IR in Tg mice could be secondary to increased obesity or the direct effects of overexpression in other cells, such as hepatocytes, and this needs to be further investigated. These findings indicate that *TRPC1* maintains glucose and insulin homeostasis and regulates lipid metabolism. Similar to those reported by Keiichiro Matoba *et al.* (42), we also found that compared with WT, obese Tg mice exhibited higher levels of leptin (an established marker of adiposity) (43, 44). However, high serum leptin levels in obese mice do not necessarily mean leptin resistance has developed; further systematic verification is required in the future.

The PI3K/AKT signaling pathway is one of the main pathways regulating adipogenic differentiation. It has been reported that the inhibition of PI3K and the loss of AKT1/protein kinase B or AKT2/protein kinase B inhibits adipogenesis. Mice lacking AKT1 and AKT2 exhibit lipoatrophy, and their cells cannot differentiate (45). The relevance of this pathway in humans is emphasized by the discovery of a family with lipoatrophic diabetes carrying an *AKT2* mutation (46). Our data show that *TRPC1* activates the PI3K/AKT pathway by upregulating the protein expression levels of p-AKT and p-PI3K in adipocytes, a phenomenon similar to that observed in colorectal cancer cells (47), human atrial myocytes (48), and diabetic rats (49).

The Wnt/β-catenin signaling pathway also plays a pivotal role in regulating preadipocyte differentiation and adipogenesis (50). It has been proven that the Wnt/β-catenin signaling pathway inhibits adipogenesis and plays an important role in maintaining the undifferentiated state of precursor adipocytes (51, 52). The activated Wnt signaling pathway leads to decreased phosphorylation of β-catenin, resulting in increased protein stability (53). We showed that *TRPC1* inhibited the Wnt/β-catenin signaling pathway by upregulating the phosphorylation level of β-catenin protein and thus playing a role in regulating adipogenesis. However, *TRPC1* did not affect the amount of total β-catenin protein expression in adipocytes, which was different from our previous results in myocytes (8), indicating that the regulation of the Wnt/β-catenin pathway by *TRPC1* is cell-specific. Krout *et al.* showed that *TRPC1* deficiency also downregulated the phosphorylation of ERK2 in subcutaneous adipose tissue (10). This finding is congruent with prior investigations in which the activation of Ca^2+^ channels in adipocytes led to an elevation in ERK2 phosphorylation (54). Whether a cross-talk exists between the ERK and two signaling pathways we discovered during the process of *TRPC1* regulating adipogenesis remains to be further investigated and verified in subsequent research. Ziemba *et al.* found that calcium flow strongly activates PI3K in leukocytes (55). Increased intracellular calcium also activates CaM-dependent protein kinase and AMP-activated protein kinase (56, 57, 58), which in turn mediates AKT activation by increasing the phosphorylation level of AKT (59, 60, 61).

Moreover, Ca^2+^ accumulation also upregulated AKT and β-catenin phosphorylation in gastric cancer cells (62). As TRPC1 is a channel protein for calcium ions, it can mediate the entry of calcium ions into various cells (9). Therefore, we speculate that *TRPC1* mediates the accumulation of calcium ions in adipocytes, activating PI3K and AKT by elevating their phosphorylation levels and promoting adipogenesis. However, this needs to be further studied and verified using pathway inhibitors.

In summary, *TRPC1* was highly expressed within the adipose tissue of pigs with a strong fat deposition ability and promoted adipocyte proliferation and lipogenic differentiation. Tg-p*TRPC1* aggravated HFD-induced obesity, hepatic fat deposition, and IR. In addition, *TRPC1* may regulate adipogenesis by increasing the phosphorylation of PI3K/AKT and β-catenin. These findings suggest that *TRPC1* is a positive regulator of fat deposition, which is conducive to a better understanding of the process of fat deposition and provides an idea for exploring its molecular mechanism.

## Data availability

The data supporting this study’s findings are available from the corresponding author upon reasonable request.

## Supplemental data

This article contains supplemental data.

## Conflict of interest

The authors declare that they have no conflicts of interest with the contents of this article.

## Supplementary data

Revised Supplemental material

Original Images for Blots
