# Supplementary material for: Porcine transient receptor potential channel 1 promotes adipogenesis and lipid deposition
Source: J Lipid Res. 2024 Dec 3;66(1):100718. doi: 10.1016/j.jlr.2024.100718 (PMC11741951; doi:10.1016/j.jlr.2024.100718)
Supplement: Revised Supplemental material [file mmc1.docx]

**SUPPLEMENTAL MATERIALS**


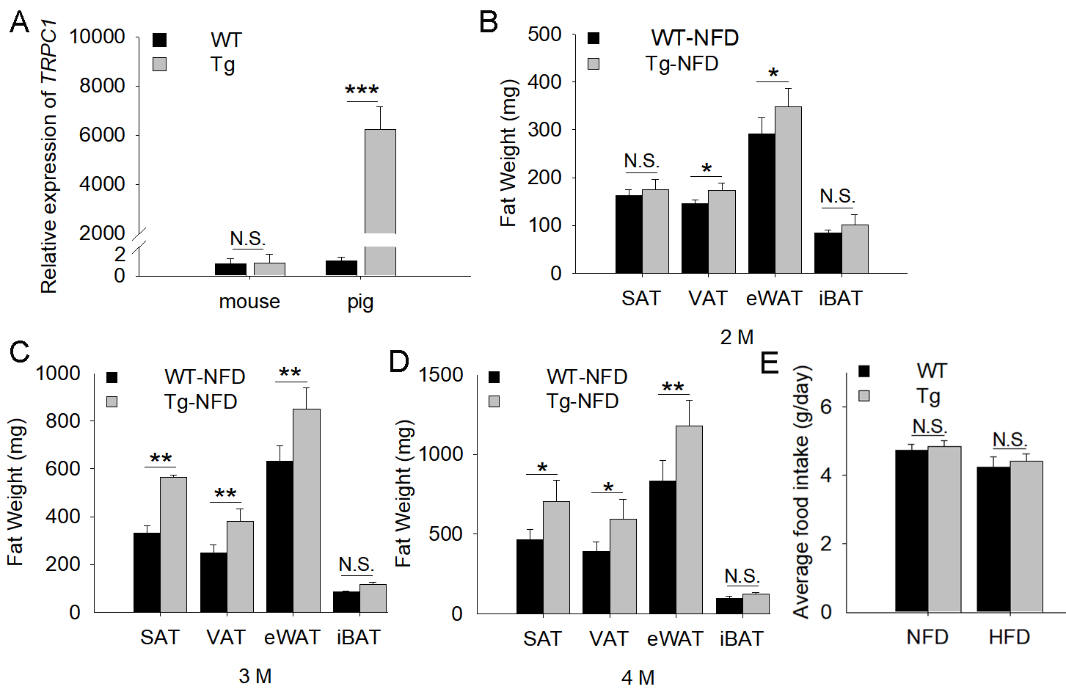


**Supplementary Figure S1 Tg-p*TRPC1* increases fat mass**

A: mRNA expression of TRPC1 in fat tissues of WT and Tg mice on normal feeding diet (NFD). B-D: Fat weight of WT and Tg mice aged from two to four months (2-4 M) on NFD. The relative mRNA levels are normalized to those of the control β-actin. E: Food intake between WT and Tg mice. SAT: subcutaneous adipose tissue; VAT: visceral adipose tissue; eWAT: epididymal white adipose tissue; iBAT: interscapular brown adipose tissue. The data represent the mean ± SD of 6 independent experiments. *: *P*<0.05, **: *P*<0.01, ***: *P*<0.001, as assessed by a student’s *t*-test. N.S.: not significant.


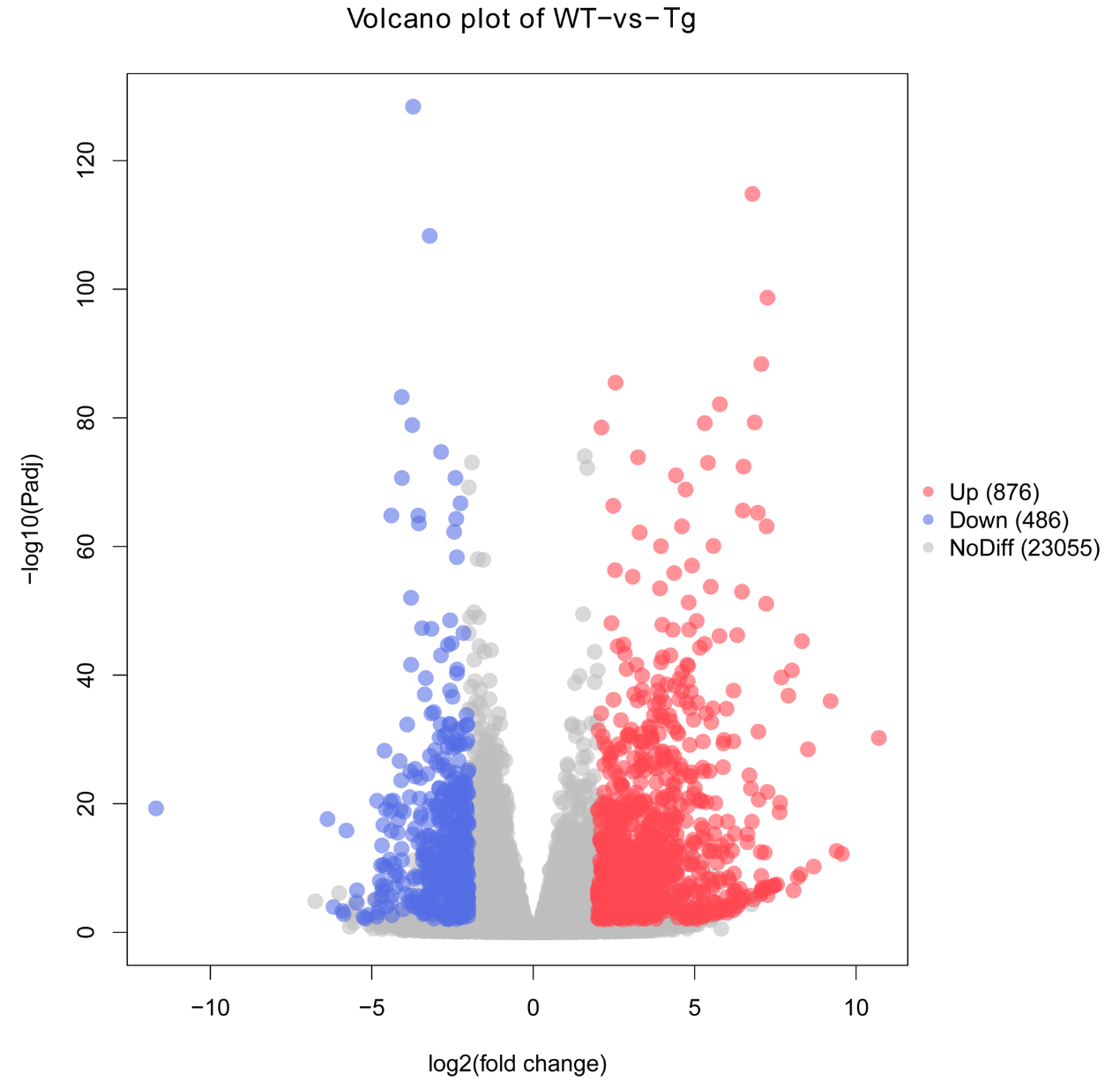


**Supplementary Figure S2 Differentially expressed genes identification**

Volcano plot displaying DEGs between WT and Tg mouse primary preadipocyte that were allowed to differentiate for 4 d. Upregulated and downregulated genes are shown in red and blue, respectively. Gray dots represent genes with similar expression levels.

**Supplementary Table S1 The primer sequences for qRT-PCR**

| Species | Genes | | Primer sequences (5′ to 3′) |
| --- | --- | --- | --- |
| Pig  Mouse | *TRPC1*  *CDK4*  *Cyclin B*  *BAD*  *PPARγ*  *CEBPβ*  *Fabp4*  *β-actin*  *PPARγ*  *CEBPα*  *CEBPβ*  *Fabp4*  *Cyclin B* | F: CATCCAAAGGCAAGGTTA  R: AAGTCCGAAAGCCAAGTA  F: CTTTGACCTGATTGGGCTGC  R: CAGAGATTCGCTTGTGTGGGT  F: TGAGGAAGAACAAGCAGTTAGACC  R: TCACAAAGGCAAAGTCACCAAT  F: TGAGCAGAGTGAGCAGGAAGAC  R: TGGGTAAGAGCTGTGGCGA  F: ACCAAAGCAAAGGCGAGG  R: GCGAAACTGACACCCCTGA  F: CCTGTCCACATCCTCGTCGT  R: CTCGTCGCTGTGCTTATCCA  F: CAGGAAAGTCAAGAGCACCA  R: GGTAGCCGTGACACCTTTC  F: GCCAACCGTGAGAAGATGACT  R: GTGACCCCATCCCCAGAGT  F: CCAAGAATACCAAAGTGCGATCA  R: CCCACAGACTCGGCACTCAAT  F: TGGACAAGAACAGCAACGAG  R: TCACTGGTCAACTCCAGCAC  F: ATCGACTTCAGCCCCTACCT  R: TAGTCGTCGGCGAAGAGG  F: AAGAAGTGGGAGTGGGCTTTG  R: CTCTTCACCTTCCTGTCGTCTG  F: ATACCTACAGGGTCGTGAAGTGA  R: GCTGTATCATCTTCTTGGGCAC | |
|  | *CDK4*  *PLIN2*  *Fasn* | F: GCTGCTACTGGAAATGCTGACC  R: AGCCTTGGGGGGAAACAGA  F: GGCTACGACGACACCGATGA  R: TAACCCTTGGGCGTTGACC  F: CACTGCATTGACGGCCGGGT  R: GGACAAGCCCAGGCTGCGAG | |
|  | *Adipoq*  *LPL*  *HSL*  *ATGL*  *TRPC1*  *β-actin* | F: GCCGCTTATGTGTATCGCTCAG  R: TTGCCAGTGCTGCCGTCATA  F: ATGCAGAAGCCCCCAGTCGC  R: GCCCCACTGGTTTCTGGATCCCA  F: CACACCTACTACACAAATCC  R: GGCATAGTAGGCCATAGCA  F: CCAACGCCACTCACATCTAC  R: CCTCAATAATGTTGGCACCTG  F: GTGGTATGAAGGGTTGGAAGACT  R: TCTGCTACAAGCGTGGGGTG  F: ATCTGGCACCACACCTTCTACA  R: AAGGTCTCAAACATGATCTGGGT | |

**Supplementary Table S2 The sequences of TRPC1 siRNA fragments**

| siRNA (pig) | Oligo sequences (5′ to 3′) |
| --- | --- |
| si-NC | UUCUCCGAACGUGUCACGUTT |
|  | ACGUGACACGUUCGGAGAATT |
| siRNA-175 | GGUGACUAUUAUAUGGUUATT |
|  | UAACCAUAUAAUAGUCACCTT |
| siRNA-339 | GGAGCGAAUUCAGAAUCCUTT |
|  | AGGAUUCUGAAUUCGCUCCTT |
| siRNA-568 | CCCGCUCUAAUAAUGUUAATT |
|  | UUAACAUUAUUAGAGCGGGTT |
| siRNA-727 | GCCCGAAAUUCACGUGAAUTT |
|  | AUUCACGUGAAUUUCGGGCTT |
