# Supplementary material for: Porcine transient receptor potential channel 1 promotes adipogenesis and lipid deposition
Source: J Lipid Res. 2024 Dec 3;66(1):100718. doi: 10.1016/j.jlr.2024.100718 (PMC11741951; doi:10.1016/j.jlr.2024.100718)
Supplement: Original Images for Blots [file mmc2.pdf]

Fig. 2H

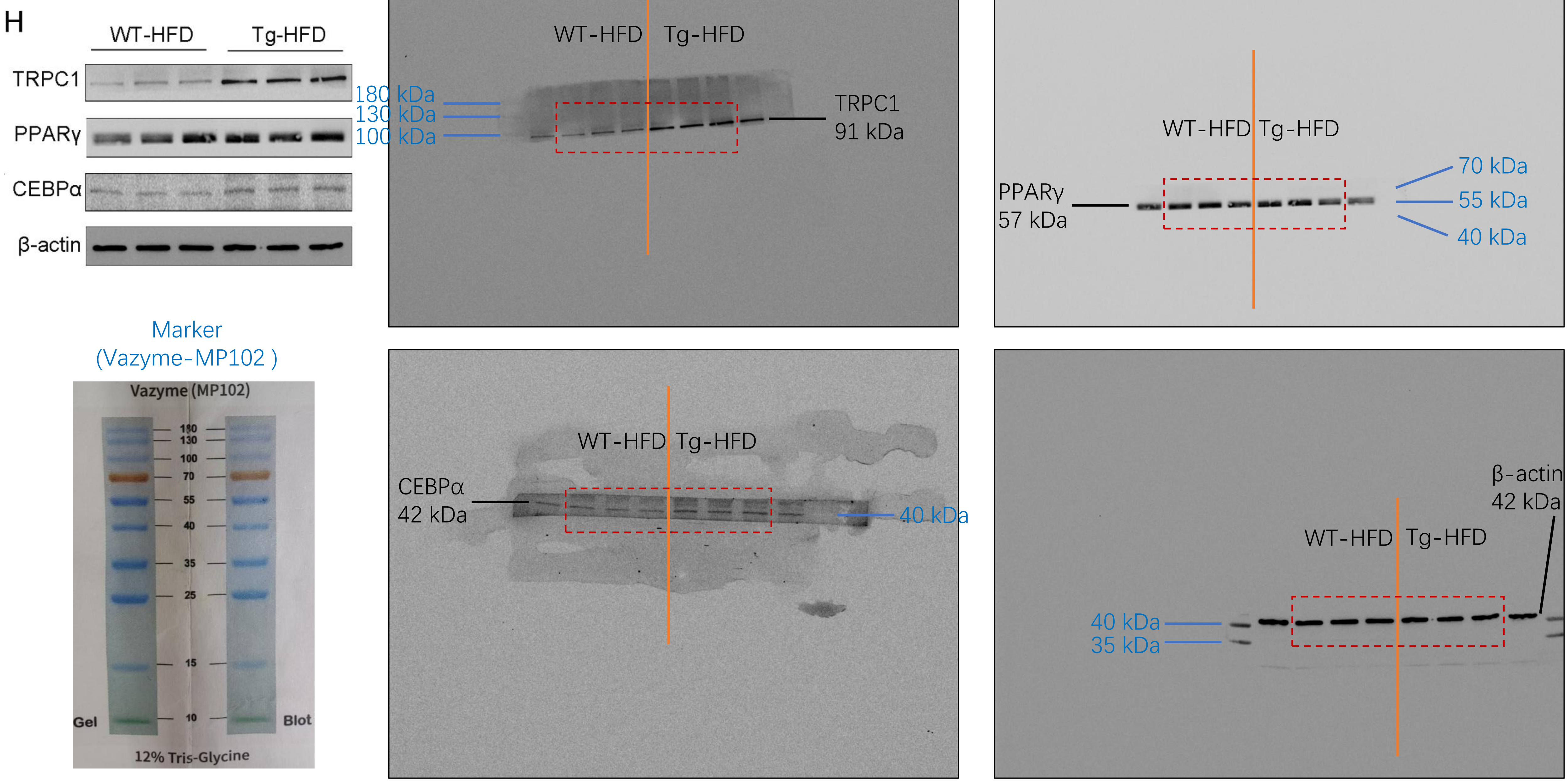
H
WT-HFD
Tg-HFD
TRPC1
PPARγ
CEBPα
β-actin
Marker
(Vazyme-MP102)
Vazyme (MP102)
180
130
100
70
55
40
35
25
15
10
Gel
Blot
12% Tris-Glycine
WT-HFD
Tg-HFD
180 kDa
130 kDa
100 kDa
TRPC1
91 kDa
WT-HFD
Tg-HFD
PPARγ
57 kDa
70 kDa
55 kDa
40 kDa
WT-HFD
Tg-HFD
CEBPα
42 kDa
40 kDa
β-actin
42 kDa
WT-HFD
Tg-HFD
40 kDa
35 kDa

Fig. 4F

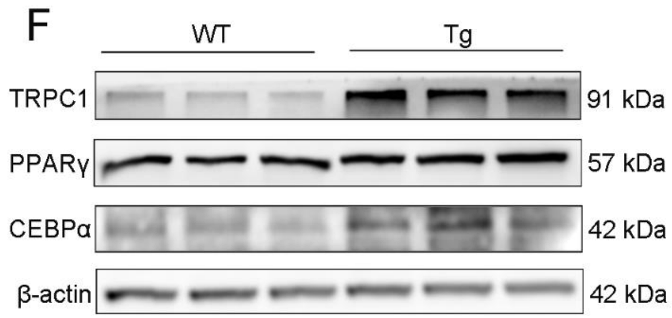

Marker
(Vazyme-MP102)

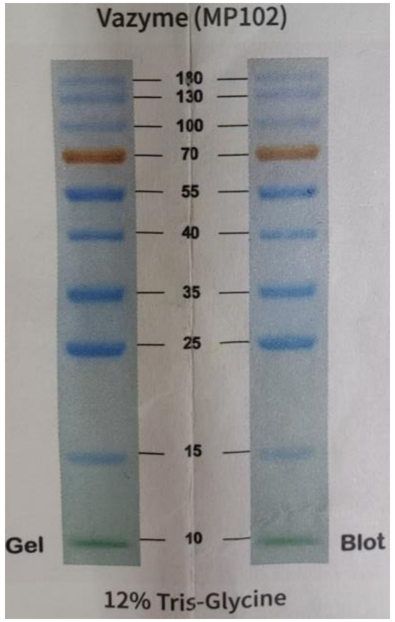

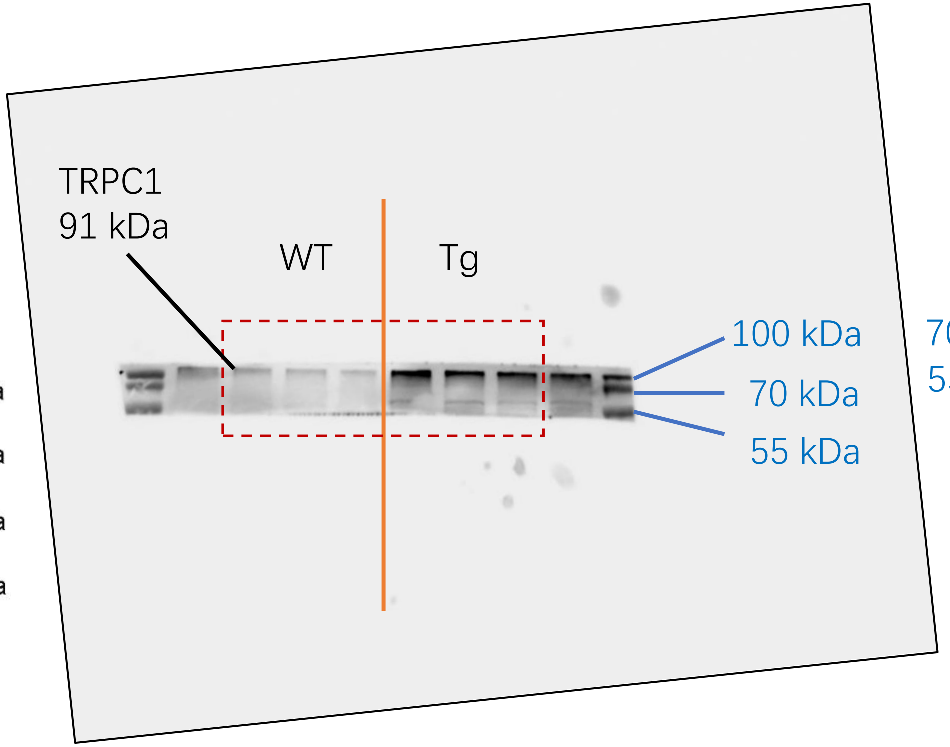

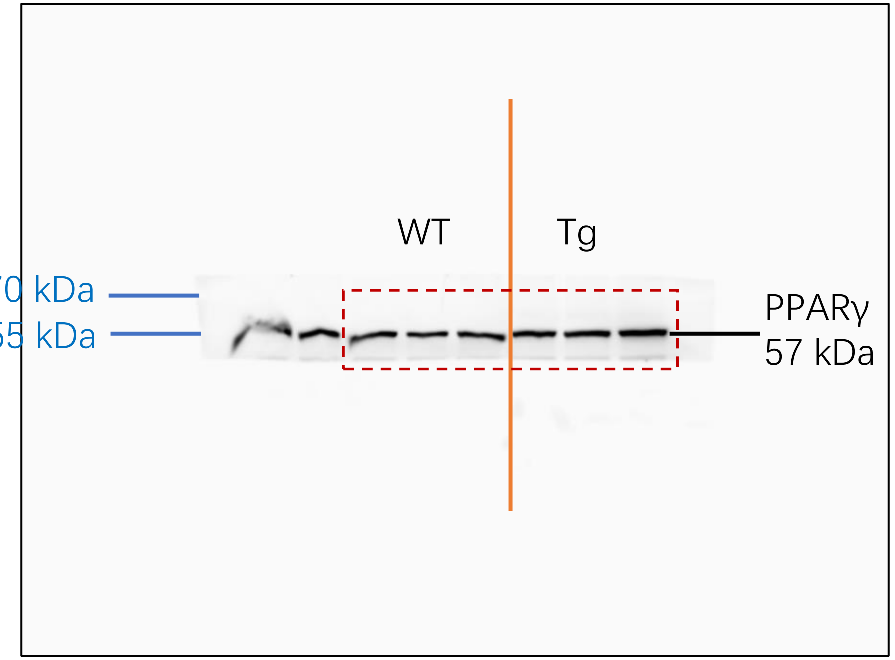

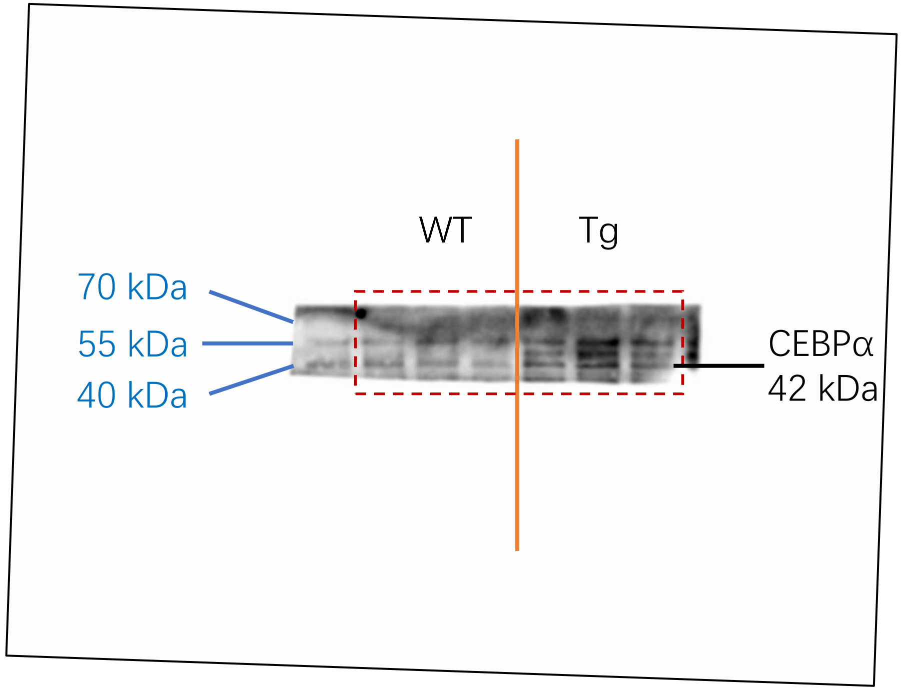

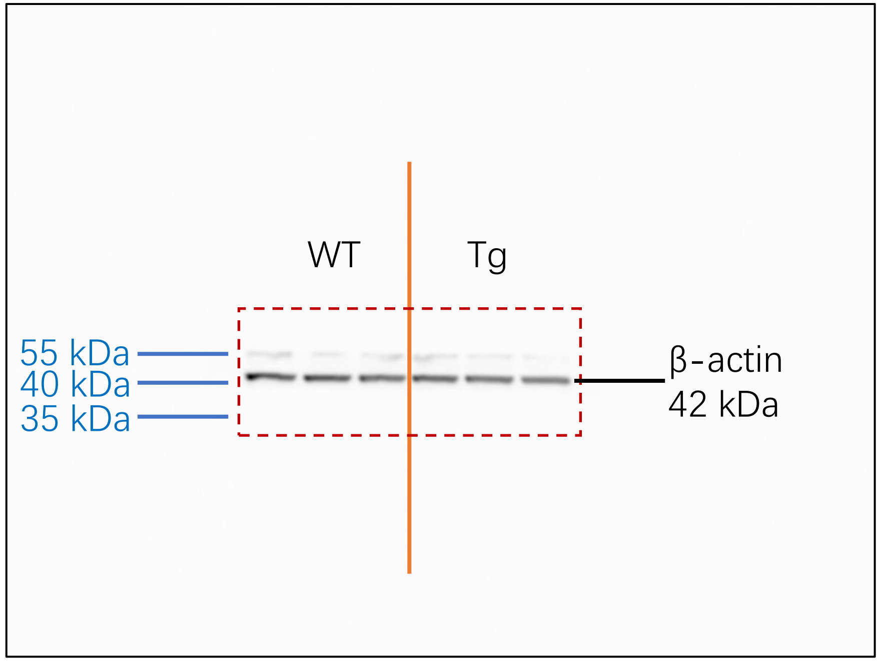

Fig. 5D

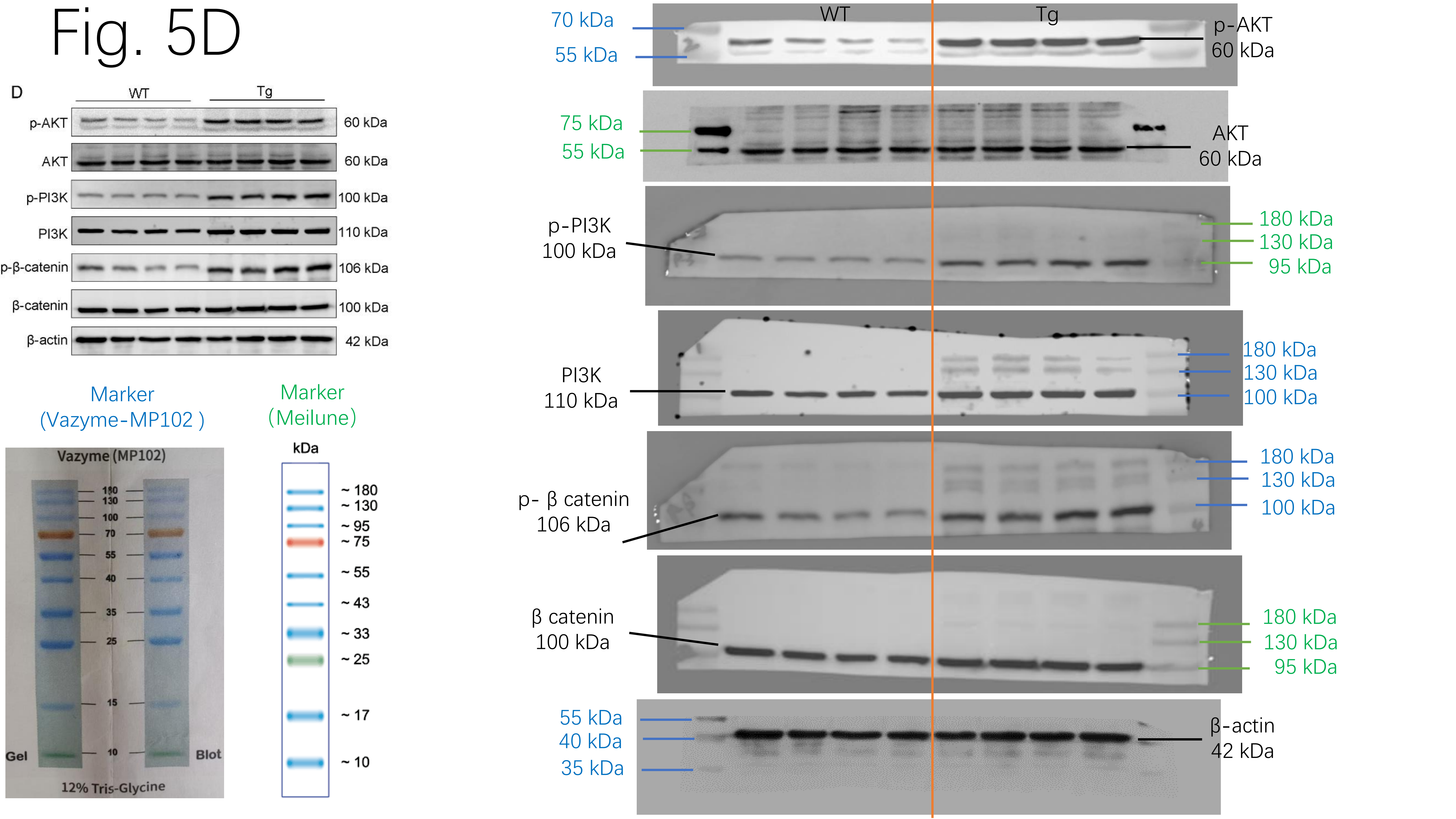

D
WT
Tg
p-AKT
60 kDa
AKT
60 kDa
p-PI3K
100 kDa
PI3K
110 kDa
p-β-catenin
106 kDa
β-catenin
100 kDa
β-actin
42 kDa
Marker (Vazyme-MP102)
Marker (Meilune)
Vazyme (MP102)
180
130
100
70
55
40
35
25
15
10
Gel
Blot
12% Tris-Glycine
kDa
~ 180
~ 130
~ 95
~ 75
~ 55
~ 43
~ 33
~ 25
~ 17
~ 10
70 kDa
55 kDa
WT
Tg
p-AKT 60 kDa
75 kDa
55 kDa
AKT 60 kDa
p-PI3K 100 kDa
180 kDa
130 kDa
95 kDa
PI3K 110 kDa
180 kDa
130 kDa
100 kDa
p- β catenin 106 kDa
180 kDa
130 kDa
100 kDa
β catenin 100 kDa
180 kDa
130 kDa
95 kDa
55 kDa
40 kDa
35 kDa
β-actin 42 kDa

Explanation for the missing of the marker：

In this manuscript, the protein markers we used were all from Meilune and Vazyme, two biological companies, and the protein band was visualized using Chemiluminescence Kit. As far as we know, the markers of these two protein markers are unable to see bands under chemiluminescence conditions. However, based on the original images we provided, it can be seen that some of the images can show the marker, and the size and position of the stripes are correct, and can correspond one-to-one with the marker manual.

The reasons are as follows:

The essence of a protein marker is a collection of proteins with different molecular weights. During the experiment, due to non-specific binding between the primary antibody of the target protein with the protein bands of the marker, marker bands appear in subsequent imaging results. However, this non-specific binding occurs by chance, occasionally under specific experimental conditions, and is not controlled by human factors. The stronger the specificity of the antibody, the lower the probability of the marker being visualized. That's why some of the original images we provide may have missing markers. During our experiment, we combined the exposed images with the position of the colored dye on the PVDF membranes of the marker (visible to the naked eye) to jointly determine the position of the target band.
